# Production of human CAR-NK cells with lentiviral vectors and functional assessment *in vitro*

**DOI:** 10.1016/j.xpro.2021.100956

**Published:** 2021-11-17

**Authors:** Ana L. Portillo, Richard Hogg, Ali A. Ashkar

**Affiliations:** 1 Department of Medicine, McMaster University, Hamilton, ON L8N 3Z5, Canada; 2 McMaster Immunology Research Centre, McMaster University, Hamilton, ON L8S 4K1, Canada

**Keywords:** Cancer, Cell Biology, Cell culture, Cell-based Assays, Flow Cytometry/Mass Cytometry, Gene Expression, Immunology, Molecular Bio

## Abstract

Although natural killer (NK) cells have become a promising immune effector cell for chimeric antigen receptor (CAR)-based therapy, generating human CAR-NK cells with high transgene efficiency has been challenging. In this protocol, we describe how to generate CAR-NK cells with transduction efficiencies >15% from healthy donor *ex vivo* expanded NK cells using third generation lentiviral vectors (LVs). We also show how to assess CAR-NK cell anti-tumor function *in vitro* using a flow cytometry-based killing assay.

For complete details on the use and execution of this protocol, please refer to Portillo et al. (2021).

## Highlights

• PBMC isolation and expansion of human NK cells from healthy donors
• Production of third generation lentiviral vectors for NK cell transduction
• A protocol for the generation of CAR-modified NK cells from human expanded NK cells
• A flow cytometry-based assay to assess CAR-NK cell cytotoxicity *in vitro*

## Graphical abstract

**Figure:**
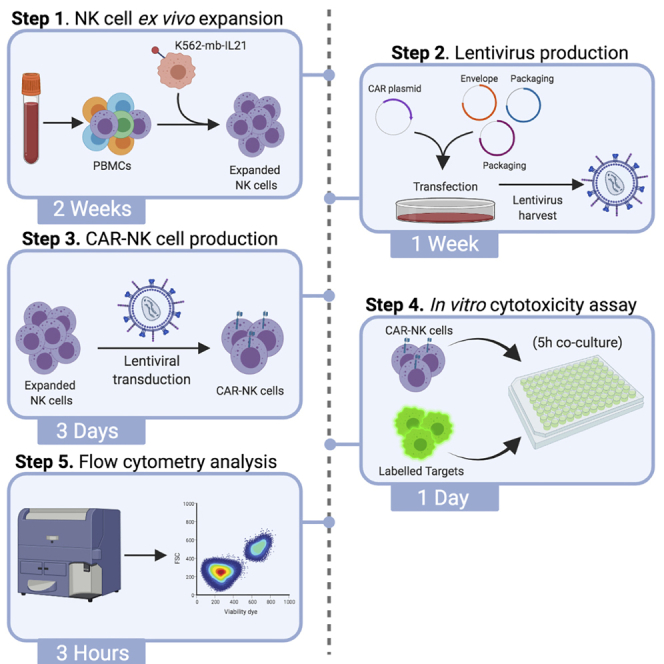

## Before you begin

***Note:*** Within each section of the protocol we have indicated when a reagent/equipment can be substituted with a similar product, otherwise use the provided reagent/equipment.

### Culture K562-mb-IL21 feeder cells

**Timing: Minimum 2 weeks before NK cell expansion from PBMCs**

This section describes how to culture the K652 feeder cells expressing membrane bound IL-21 (K562-mb-IL21) that are used to generate expanded NK cells. The K562-mb-IL21 cells were generated by retroviral transduction with truncated CD19, CD64, CD86 and CD137L and by transduction using the *Sleeping Beauty* transposon expressing mbIL-21 (Denman et al., 2012; Singh et al., 2011). The sequence of mb-IL21 is owned by Kiadis Pharma (https://www.kiadis.com/) and the sequence is expressed from the MNDU3 promoter (Singh et al., 2011). The K562-mb-IL21 cells are available upon request through a Material Transfer Agreement (MTA).

1. Thaw cryopreserved K562-mb-IL21 cells.
  a. Add 10 mL of complete RPMI media to 15 mL falcon tube.
  b. Remove frozen K562-mb-IL21 cryovial from liquid nitrogen tank.
  c. Place K562-mb-IL21 cryovial in a 37°C water bath immediately after retrieving from liquid nitrogen tank. As soon as there is one ice crystal remaining, transfer cells drop wise into 10 mL of complete RPMI media.
  d. Centrifuge tube at 300 × *g* for 5 min.
  e. Remove supernatant and count cells with Trypan Blue.
2. Resuspend K562-mb-IL21 cells to a final concentration of 0.5 × 10^6^ cells/mL in complete RPMI media. Incubate cells at 37°C and 5% CO_2_
3. Change media every 2–3 days and maintain cells at 0.5 × 10^6^ cells/mL.

### Isolation of peripheral blood mononuclear cells (PBMCs) from whole blood

Obtain approval from the appropriate ethics board prior to conducting research with human samples. All research conducted in this study was approved by the Hamilton Integrated Research Ethics Board in Hamilton, Ontario. Before taking blood ensure that you have obtained written and signed consent from the healthy donors. Follow all appropriate safety guidelines when working with human blood including use of a biological safety cabinet.

**Timing: 2 h**

4. Prepare 50 mL falcon tubes containing 15 mL of Lymphoprep density gradient medium.

***Alternatives:*** Other density gradient media suitable for PBMC isolation can be used such as Ficoll.

5. Collect 15–30 mL of whole blood from a healthy donor using BD ACD Solution A Vacutainers.
6. Transfer 15 mL of peripheral blood per 50 mL falcon tube.
7. Use 15 mL of sterile 2% FBS-PBS to wash vacutainer tubes and dilute the peripheral blood 1:1 in the 50 mL falcon tube.
8. Set your pipettor to slow speed and begin slowly overlaying the 30 mL of diluted peripheral blood onto the falcon tube pre-filled with Lymphoprep.

**CRITICAL:** Make sure the diluted blood does not break through the Lymphoprep layer to avoid red blood cell contamination after centrifugation. Tilt the tube and allow the blood to flow down the tube wall.

**Figure 1:**
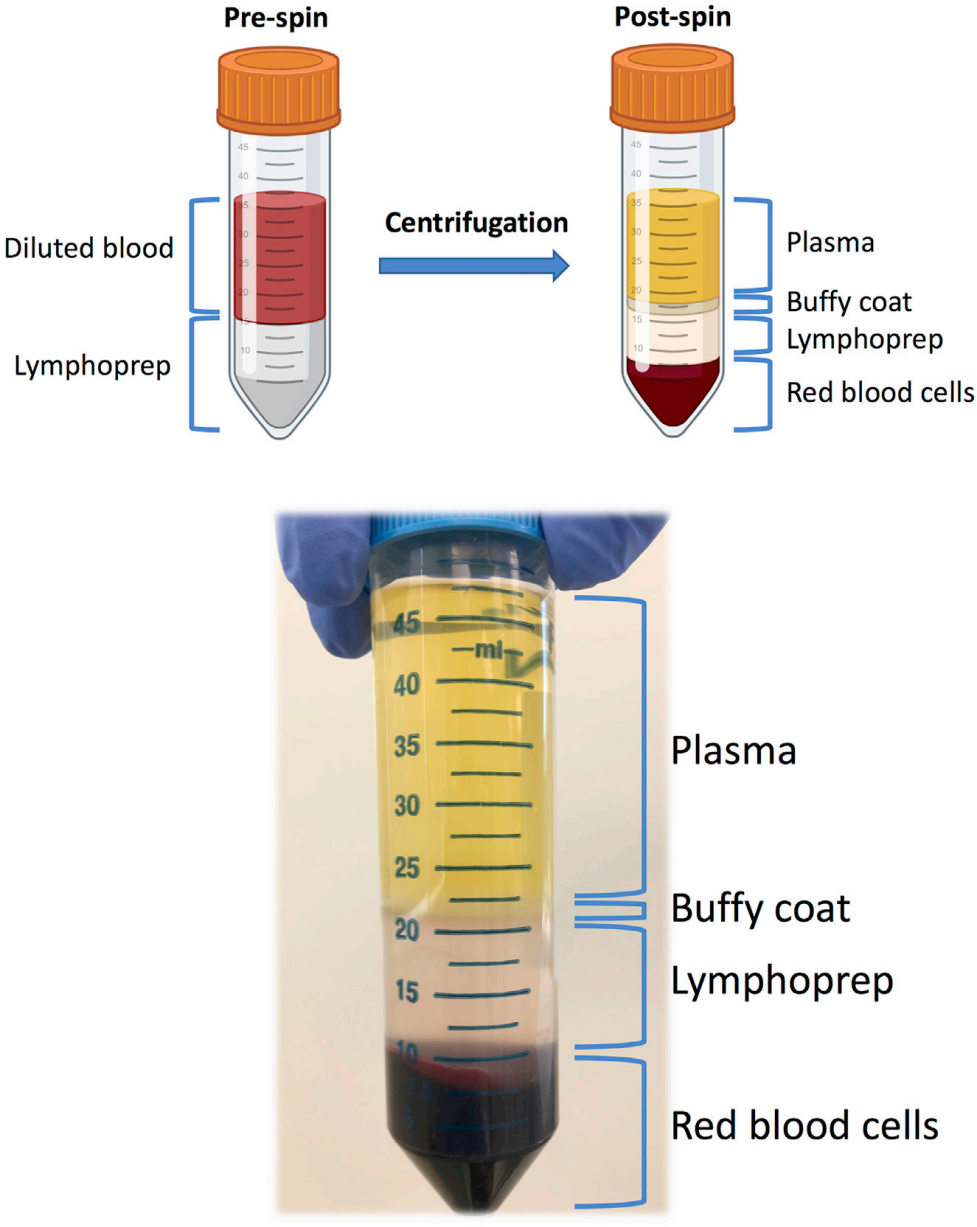
Schematic of the different blood layers obtained from the density gradient centrifugation pre- and post-spin.
10. Slowly remove ∼10 mL of the plasma layer without disturbing the buffy coat and discard.
11. Slowly collect the buffy coat and place into a new 50 mL falcon tube being careful to not disturb the red blood cell pellet at the bottom of the tube.

***Note:*** Buffy coats collected from two tubes can be combined into one 50 mL falcon tube.

9. Centrifuge at 20°C–22°C for 20 min at 600 × *g* with no brakes. See Figure 1 for visual representation of blood component layers after centrifugation.

12. Top up the tube with 2% FBS-PBS and centrifuge at 300 × *g* for 5 min with the break on to remove remaining Lymphoprep.
13. Discard supernatant and count isolated PBMCs with Trypan Blue.

**Pause point:** At this point you can proceed directly to the *ex vivo* NK cell expansion using fresh PBMCs or cryopreserve them for later use.

### *Ex vivo* expansion of human NK cells using K562-mb-IL21 feeder cells

**Timing: Minimum 2 weeks**

This section describes how to generate highly activated CD3^-^CD56^superbright^ expanded NK cells from human PBMCs.

14. Transfer K562-mb-IL21 cells to a 50 mL falcon tube and centrifuge at 300 × *g* for 5 min.
  a. Remove supernatant and count with Trypan Blue.
  b. Determine total number of K562-mb-IL21 cells needed to replenish desired number of PBMCs at a 2:1 ratio.

Example: If expanding a starting number of 2.5 million PBMCs you need to irradiate 5 million K562-mb-IL21 cells. Depending on the number of NK cells you would like to expand, irradiate the corresponding amount of K562-mb-IL21 plus 2–5 million extra cells to account for pipetting error.

15. Resuspend K562-mb-IL21 cells to 5 × 10^6^ cells/mL in complete RPMI media and irradiate cells using a gamma cell irradiator at 100 Gy for 30 min.
16. Co-culture irradiated K562-mb-IL21 feeder cells with PBMCs at a 2:1 ratio, respectively.
  a. Count PBMCs with Trypan Blue.
  b. Add 2.5 million PBMCs and 5 million irradiated K562-mb-IL21 cells into a 50 mL falcon tube and top up with complete RPMI media to a final volume of 0.5 × 10^6^ cells/mL based on the starting PBMC cell number.
  c. Supplement with 100 IU/mL of IL-2.
17. Incubate the co-culture at 37°C and 5% CO_2._

***Note:*** Starting with 2.5 million PBMCs usually yields a sufficient amount of expanded NK cells as this method typically induces a ∼10^3^–10^4^ fold expansion after 21 days (Denman et al., 2012).

18. Replace media on co-culture every other day.
  a. Transfer co-culture to a 50 mL falcon tube and centrifuge at 300 × *g* for 5 min.
  b. Remove supernatant and add 5 mL of fresh complete RPMI media, keeping the same total volume used in the replenishing day.
  c. Supplement with 100 IU/mL of IL-2.
  d. Place co-culture back into tissue culture flask and incubate at 37°C and 5% CO_2._
19. Every 7 days, count NK cells in the culture and add freshly irradiated K562-mb-IL21 cells at a 2:1 ratio. See Troubleshooting 1 if low viability or low expansion is observed.

***Note:*** Prior to proceeding with lentiviral transduction, ensure that the expanded NK cells are >75% CD3-CD56^superbirhgt^ (Troubleshooting 2).

### Culture SKBR3 breast cancer tumor cells

**Timing: Minimum 1 week before *in vitro* cytotoxicity assays**

The following protocol describes how to passage the human breast cancer SKBR3 cell line established from a 43-year old Caucasian female with malignant mammary adenocarcinoma and which naturally overexpresses HER2. Other target cells can be used depending on the specificity of the CAR construct used in your study.

20. Thaw cryopreserved SKBR3 cells.
  a. Add 10 mL of complete DMEM media to 15 mL falcon tube.
  b. Remove frozen SKBR3 cryovial from liquid nitrogen tank.
  c. Place SKBR3 cryovial in a 37°C water bath immediately after retrieving from liquid nitrogen tank. As soon as there is one ice crystal remaining, transfer cells drop wise into 10 mL of complete DMEM media.
  d. Centrifuge tube at 300 × *g* for 5 min.
  e. Remove supernatant and count cells with Trypan Blue.
21. Resuspend 2.5–5 million SKBR3 cells in 20 mL of complete DMEM and place in a T-150 flask.
22. Passage cells every 2–3 days once they reach confluency.
  a. Discard spent media from flask. Add 10 mL of warm PBS to wash the cells.
  b. Add 5 mL of 1× Trypsin-EDTA (0.05%) and incubate at 37°C for 3–5 min.
  c. Lightly tap flask to dislodge all cells from the flask.
  d. Neutralize Trypsin by adding 3 times the volume of warm complete DMEM media.
  e. Split confluent flask by 1:4.

## Key resources table

**Table:** 

REAGENT or RESOURCE	SOURCE	IDENTIFIER
**Antibodies**		

PE Mouse Anti-Human IgG Fc (1:20)	BioLegend	Cat#409304; Clone HP6017; RRID:AB_10895907
APC Mouse Anti-Human CD271 (NGFR) (1:20)	BioLegend	Cat#345108; Clone ME20.4; RRID:AB_10645515
BV421 Mouse Anti-Human CD56 (1:20)	BD Biosciences	Cat#562751; Clone NCAM16.2; RRID:AB_2732054
APC-H7 Mouse Anti-Human CD3 (1:20)	BD Biosciences	Cat#560275; Clone SK7; RRID:AB_1645475
VioBright FITC Mouse Anti-Human CD271 (LNGFR) (1:50)	Miltenyi Biotec Inc.	Cat#130-113-423; Clone ME20.4-1.H4; RRID:AB_2734064

**Bacterial and virus strains**		

Third Generation Self-Inactivating Lentivirus	Generated in this study	N/A

**Biological samples**		

Healthy adult PBMCs	Isolated from peripheral blood of healthy volunteers	N/A

**Chemicals, peptides, and recombinant proteins**		

Lymphoprep	STEMCELL Technologies	Cat#07861
Recombinant Human IL-2	PeproTech	Cat#200-02; Accession# P60568
Recombinant Human ErbB2/Her2 Fc Chimera Protein	R&D Systems	Cat#1129-ER-050; Accession# NP_004439
Hexadimethrine bromide (Polybrene)	Sigma-Aldrich	Ca# H9268; CAS: 28728-55-4
5(6)-Carboxyfluorescein diacetate N-succinimidyl ester (CFSE)	Sigma-Aldrich	Cat#21888; CAS 150347-59-4
eBioscience Fixable Viability Dye eFluor 780 (1:1000)	Thermo Fisher Scientific	Cat#65-0865-18
Fixable Viability Stain 510 (1:1000)	BD Biosciences	Cat#564406; RRID: AB_2869572
Lipofectamine 2000	Thermo Fisher Scientific	Cat#11668019
Sodium butyrate	Sigma-Aldrich	Cat#B5887; CAS: 156-54-7
Opti-MEM	Thermo Fisher Scientific	Cat#31985070
Fetal Bovine Serum	Gibco	Cat#12484028
Bovine Serum Albumin	Rockland Inc.	Cat#BSA-50
RMPI 1640 Medium, powder	Gibco	Cat#31800105
DMEM Medium, powder	Gibco	Cat#12800017
L-Glutamine	Bioshop Canada Inc	Cat#GLU102; CAS: 56-85-9
HEPES	In house	N/A
Penicillin/Streptomycin	Gibco	Cat#15140122
Trypan Blue Solution, 0.4%	Life Technologies	Cat#15250061
Dimethylsulfoxide (DMSO)	Sigma-Aldrich	Cat#D12345
10× Trypsin-EDTA (0.5%)	Gibco	Cat#15400054
16% Paraformaldehyde (PFA)	Electron Microscopy Sciences	Cat#15710(EM)

**Experimental models: cell lines**		

Human: K562-mb-IL21 (Clone 9) cells (Female)	Laboratory of Dean A. Lee	Denman et al. (2012)
Human: SKBR-3 cells (Female)	Laboratory of Karen Mossman	RRID: CVCL_0033
Human: HEK293T cells (Fetal)	Laboratory of Jonathan L. Bramson	RRID: CVCL_0063

**Recombinant DNA**		

Plasmid: pCCL-Darpin-hCD8a NGFR	Laboratory of Jonathan L. Bramson	Hammill et al. (2015)
Plasmid: pCCL-CMV-NGFR	Laboratory of Jonathan L. Bramson	Hammill et al. (2015)
Plasmid: pRSV-REV	Addgene	Addgene #12253
Plasmid: pMD2.G	Addgene	Addgene #12259
Plasmid: pMDLg-pRRE	Addgene	Addgene #12251

**Software and algorithms**		

FACSDIVA Software	BD Biosciences	https://www.bdbiosciences.com/
FlowJo Software	BD Biosciences	https://www.flowjo.com/

**Other**		

BD Vacutainer™ Glass Blood Collection Tubes with Acid Citrate Dextrose (ACD)	Thermo Fisher Scientific	Cat#02-684-26
150 mL 0.45 μM polyenersulfone (PES) filter	Thermo Fisher Scientific	Cat#165-0045
38.5 mL Ultracentrifuge tubes	Beckman Coulter	Cat#344058
SW-32 Ti Swinging Bucket Rotor	Beckman Coulter	Cat#369694
T-150 tissue culture flasks	Fisher Scientific	Cat#0877248
15 cm NUNC plates	Thermo Fisher Scientific	Cat#168381
5 mL polysterene tubes	Fisher Scientific	Cat#149591A
50 mL falcon tubes	Falcon	Cat#352098
15 mL falcon tubes	Falcon	Cat#352096
96-well U bottom plate with lid	Fisher Scientific	Cat#0877254
24-well Tissue culture plate with lid	Falcon	Cat# 353047
20 μL Sterile filtered pipette tips	DiaMed	Cat#DIATEC530-8001
100 μL Sterile filtered pipette tips	DiaMed	Cat#DIATEC530-8251
1000 μL Sterile filtered pipette tips	DiaMed	Cat#DIATEC530-9001
5 mL serological pipettes	Fisher Scientific	Cat#1367522
10 mL serological piptettes	Fisher Scientific	Cat# 1367520
25 mL serological pipettes	Fisher Scientific	Cat# 136682
1.5 mL microcentrifuge tubes	Fisher Scientific	Cat# 05408129
LSRFortessa Flow Cytometer	BD Biosciences	N/A

## Materials and equipment

**Table:** Complete RPMI media

Reagent	Final concentration	Amount
RPMI 1640 media	n/a	435 mL
FBS	10%	50 mL
HEPES (1 M)	10 mM	5 mL
L-glutamine (0.2 M)	2 mM	5 mL
Penicillin/Streptomycin	1%	5 mL
**Total**	**n/a**	**500 mL**

[Keep sterile and store at 4°C for up to 1 month]

**Table:** Complete DMEM media

Reagent	Final concentration	Amount
DMEM media	n/a	435 mL
FBS	10%	50 mL
HEPES (1 M)	10 mM	5 mL
L-glutamine (0.2 M)	2 mM	5 mL
Penicillin/Streptomycin	1%	5 mL
**Total**	**n/a**	**500 mL**

[Keep sterile and store at 4°C for up to 1 month]

**Table:** 1× Trypsin EDTA (0.05%)

Reagent	Final concentration	Amount
PBS	n/a	90 mL
10× Trypsin-EDTA (0.5%)	1×	10 mL
**Total**	**n/a**	**100 mL**

[Sterile filter and store at 4°C for up to 4 months]

**Table:** Polybrene stock

Reagent	Final concentration	Amount
Sterile Water	n/a	20 mL
Hexadimethrine bromide (Polybrene)	8000 μg/mL	0.160 g
**Total**	**n/a**	**20 mL**

[Sterile filter and store at −80°C for up to 4 months]

**Table:** NK cell transduction media

Reagent	Final concentration	Amount
Complete RPMI media	n/a	10 mL
Hexadimethrine bromide (Polybrene; 8000 μg/mL)	8 μg/mL	10 μL
IL-2 (100 IU/uL)	500 IU/mL	50 μL
**Total**	**n/a**	**10 mL**

[Make fresh each time]

**Table:** 5(6)-Carboxyfluorescein diacetate N-succinimidyl ester (CFSE) stock

Reagent	Final concentration	Amount
DMSO	n/a	896.9 μL
CFSE	50 mM	25 mg
**Total**	**n/a**	**896.9 μL**

[Store aliquots at −20°C for up to 12 months]

**Table:** Recombinant ErbB2/HER2 Fc chimeric protein stock

Reagent	Final concentration	Amount
PBS	n/a	500 μL
ErbB2/HER2 Fc chimeric protein	100 μg/mL	50 μg
**Total**	**n/a**	500 μL

[Keep sterile and store aliquots at −20°C for up to 3 months]

**Table:** FACS buffer

Reagent	Final concentration	Amount
PBS	n/a	500 mL
Bovine Serum Albumin	0.2%	1 g
**Total**	**n/a**	**500 mL**

[Sterile filter and store at 4°C for up to 2–3 months]

**Table:** 2% PFA

Reagent	Final concentration	Amount
PBS	n/a	7 mL
16% Paraformaldehyde (PFA)	2%	1 mL
**Total**	**n/a**	**8 mL**

[Store at 4°C for up to 2 weeks protected from light]

**CRITICAL:** 16% PFA is classified as hazardous under WHIMIS 2015. Handle inside a fume hood and use double gloves. Immediately rinse with water if there is contact with the skin. Dispose in appropriate chemical waste container.

## Step-by-step method details

### Third generation lentivirus production and harvest by ultracentrifugation

**Timing: 1 week**

These steps describe how to generate 3^rd^ generation LVs and has been adapted with some modifications from Hammill et al. (2016).

1. Thaw low passage (P16 or less) cryopreserved HEK293T cells in a 37°C water bath.
  a. Add 10 mL of complete DMEM media to 15 mL falcon tube.
  b. Remove frozen HEK293T cryovial from liquid nitrogen tank.
  c. Place HEK293T cryovial in a 37°C water bath immediately after retrieving from liquid nitrogen tank. As soon as there is one ice crystal remaining, transfer cells drop wise into the 15 mL falcon tube.
  d. Centrifuge tube at 300 × *g* for 5 min.
  e. Remove supernatant and count cells with Trypan Blue.
2. Resuspend 1.5–2 million HEK293T cells in 20 mL of complete DMEM media and transfer to a T-150 tissue culture flask. Culture flasks at 37°C and 5% CO_2_.

***Note:*** Always start lentivirus prep with freshly thawed HEK293T cells to ensure higher viral yield. Around 2–3 80% confluent T-150 flasks are needed for one batch of lentiviral prep.

3. 24 h prior to transfection, harvest HEK293T cells by washing flasks with 10 mL warm PBS and adding 3 mL warm 1× Trypsin-EDTA(0.05%). Place flasks in the incubator for 2–5 min and neutralize trypsin with 3× the volume of warm complete DMEM media.

***Note:*** Cells should be ∼80% confluent three days post-thaw. HEK293T cells are very loosely adherent thus do not require a long incubation time with Trypsin. Use Trypsin to harvest cells to prevent cell clumping.

4. Transfer cell suspension to a 50 mL falcon tube and centrifuge at 300 × *g* for 5 min. Resuspend cell pellet with warm complete DMEM media and count cells using Trypan Blue.

***Note:*** Use antibiotic-free complete DMEM media to plate cells prior to transfection as it may interfere with Lipofectamine 2000 reagent.

5. Plate 9 million HEK293T cells in 15 mL of antibiotic-free complete DMEM media per 15 cm NUNC tissue-culture treated plate. Incubate plates at 37°C and 5% CO_2_.
  a. Re-seed a T-150 flask with 1.5–2 million cells in 20 mL of complete DMEM media for lentiviral titration.

***Note:*** Typically, three 15 cm plates are required to obtain sufficient viral particles per prep, scale up as needed. Carefully place plates into incubator ensuring that plates are even to make sure media covers each plate evenly.

6. Transfection can begin 24 h after plating HEK293T cells, at this point cells should be ∼70%–80% confluent.

***Note:*** Measure concentration of all plasmids used prior to transfection using a spectrophotometer that measures the concentration and purity of nucleic acids.

7. For each plate, prepare two 15 mL falcon tubes with 4 mL of Opti-MEM media labeled Tube A and Tube B.
8. Prepare transfection mixture by following schematic in Figure 2.

**Figure 2:**
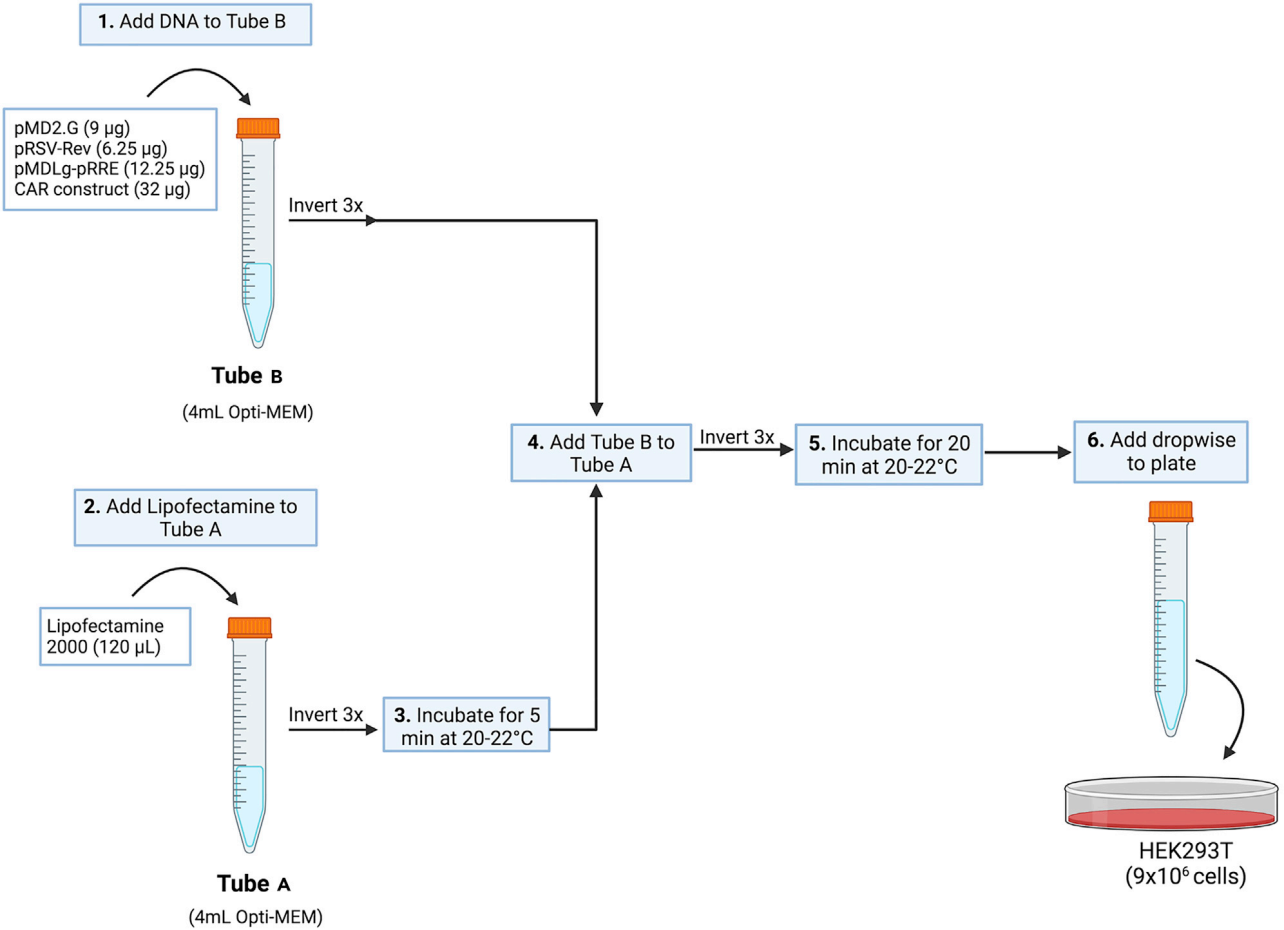
Flowchart depicting how to prepare transfection mixture (1) Add required amount of plasmid DNA to Tube B and mix gently by inverting tube 3 times (3×). (2) Add 120 uL of Lipofectamine 2000 to Tube A and mix gently by inverting tube 3 times. (3) Incubate Tube A for 5 min at 20°C–22°C. (4) Add Tube B to Tube A, mix gently by inverting tube 3 times. (5) Incubate for 20 min at 20°C–22°C. (6) Add transfection mixture to each plate.
9. Slowly add the DNA and Lipofectamine mixture to each plate drop wise making sure to cover the entire plate area. Swirl plate carefully and slowly to evenly distribute the mixture.
10. Incubate plates at 37°C and 5% CO_2_.

***Alternatives:*** Lipofecatamine (lipid based transfection agent) can be substituted with chemical methods such as calcium phosphate for the transfection. However, the amount of total DNA added should be optimized using this method. Refer to Brown et al. (2020) and Follenzi and Naldini (2002) for protocols on LV production using calcium phosphate.

11. 12–16 h after transfection, carefully aspirate media and replace with 15 mL warm antibiotic-containing complete DMEM media supplemented with 1 mM of sodium butyrate.

***Note:*** Sodium butyrate is added as it has been shown to enhance HIV-1 derived vector production (Olsen and Sechelski, 1995).

**CRITICAL:** During this step, there may be lift off the HEK293T cells from the plate which can decrease viral yield. To avoid this ensure that DMEM media is warmed up to 37°C and aspirate media as slowly as possible. Only work with up to three plates at a time.

12. Incubate plates at 37°C and 5% CO_2_.

***Note:*** From this point forward, you will be working with virus samples. Ensure you are following proper BSL-2 guidelines when handling lentivirus.

13. 36–48 h after transfection, lentivirus released in the supernatant can be harvested for viral concentration.

**CRITICAL:** In our experience, the use of ultracentrifugation to concentrate the virus cannot be replaced with other methods of viral concentration such ultrafiltration. We have found that viral preps obtained from ultrafiltration do not efficiently transduce NK cells.

***Note:*** Pre-cool the SW-32 Ti ultracentrifuge rotor and buckets at 4°C before starting the harvest.

14. Aspirate viral containing supernatant from the 15 cm plates and transfer to 50 mL falcon tubes.
15. Centrifuge the 50 mL falcon tubes at 2000 × *g* for 10 min at 4°C to sediment any collected cells.
16. Filter supernatant using a 150 mL 0.45 μM PES filter to remove cell debris.

***Alternatives:*** A filter with a pore size of 0.22 μM can also be used to improve purity. However, a size of 0.45 μM is used in this protocol to avoid any loss of viral particles during the filtration.

17. Add filtrate into 38.5 mL ultracentrifuge tubes and use cold PBS to bring tube within 0.5 cm of the edge to prevent centrifuge tube from collapsing.
18. Carefully balance the centrifuge tubes before placing into the ultracentrifuge buckets.

**CRITICAL:** Ultracentrifuge buckets containing tubes must be balanced within **0.02*g*.**

19. Centrifuge buckets at 130,000 × *g* for 1 h 40 min at 4°C with minimal deceleration.
20. Carefully remove ultracentrifuge tube from ultracentrifuge bucket and pour the supernatant into a 20% bleach bucket.
21. Keep the ultracentrifuge tube inverted and blot the excess supernatant onto paper towels for up to 1 min to prevent viral pellet from drying out.
22. Immediately place the ultracentrifuge tube on ice and let it sit for 10 min to begin viral resuspension.

**CRITICAL:** Avoid the formation of bubbles during resuspension of the viral pellet. Keep virus on ice at all times until placing in the −80°C freezer.

***Note:*** From this point forward only use micropipette filter tips when handling the virus.

23. Add between 50–100 μL of cold PBS and start to slowly scrape the bottom of the ultracentrifuge tube using a micropipette filter tip.

***Note:*** Set the micropipette to a low volume (∼40 μL) and do not go to the second stop while resuspending to avoid formation of bubbles.

***Note:*** The volume of PBS added to resuspend the viral pellet can be adjusted depending on your experience with the particular LV. For example, you can add a higher volume of PBS to lentivirus preps that have higher yields. If you consistently obtain a low viral yield see Troubleshooting 3.

24. Using the same micropipette filter tip, slowing resuspend the pellet by gently pipetting up and down and wash the sides of the ultracentrifuge tube.
25. Once viral pellet is fully resuspended aliquot virus into 1.5 mL microcentrifuge tubes and immediately place into −80°C freezer.
  a. Prepare one aliquot of 13 μL to use for the lentiviral titration.
26. Decontaminate anything that has come into contact with the virus using 70% ethanol.

***Note:*** Minimize the production of aerosols when working in BSL2 conditions.

### Lentivirus titration on HEK293T cells

**Timing: 4 days; variable flow time**

The lentivirus stock should be titrated using HEK293T cells prior to NK cell transduction. To determine the titer, HEK293T cells are first transduced with serial dilutions of the lentivirus stock and they are then stained to measure the expression of the transduction marker by flow cytometry. The titer is calculated using the following formula: Titer (TU/mL)= ((# of HEK293T cells transduced × % Transgene-positive cells × Dilution factor)/100). The expected titer for the lentivirus containing the HER2 CAR construct used in this study is between 3 × 10^7^–2 × 10^8^ TU/mL.

**CRITICAL:** The lentiviral constructs used in this study contain the NGFR transduction marker. Lentivirus with a titer >3 × 10^7^ TU/mL is necessary for NK cell transduction (Troubleshooting 4).

27. Harvest HEK293T cells from a T-150 flask and count using Trypan Blue.

***Note:*** Perform two separate counts and take the average to ensure the cell count is accurate.

**CRITICAL:** The titration is dependent on accurately plating 30,000 cells per well.

28. Plate 30,000 HEK293T cells per well in a 24-well tissue culture plate.
  a. Resuspend cells to 6 × 10^4^ cells/mL using complete DMEM media.
  b. Up to 6-wells will be needed for each lentivirus batch, using dilutions 10^−2^ to 10^−6^ and one control well with no virus.
  c. Resuspend the cell suspension 3 times and add 500 μL per well.
29. Incubate plate at 37°C and 5% CO_2_ for at least 3 h to ensure HEK293T cells have adhered.
30. Prepare lentivirus serial dilutions following Figure 3.
  a. Prepare dilutions using 1.5 mL microcentrifuge tubes in complete DMEM media.
  b. Mix each dilution thoroughly by pipetting or vortexing.

**Figure 3:**
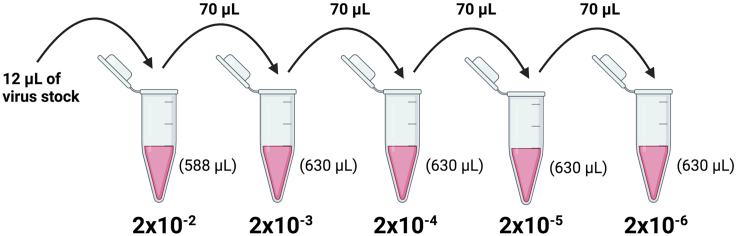
Flowchart of serial dilutions for stock lentivirus titration on HEK293T cells.
31. Thoroughly mix and add 500 μL of each dilution per well.
32. Add 500 μL of complete DMEM media to the no virus control well.
33. Incubate plate at 37°C and 5% CO_2_ for 72 h.

***Alternatives:*** We find that assessing transgene expression after 72 h post HEK293T cell transduction is adequate to obtain a determination of LV titer. However, if a highly accurate titer is needed waiting 5 days post transduction could minimize the proportion of HEK293T cells that express the transgene but has not been successfully integrated.

34. Carefully harvest the transduced HEK293T cells using 1000 μL micropipette filter tips.
  a. Slowly pipette up and down first in the centre of each well and then around the edges.
35. Transfer cells to 5 mL polysterene tubes with caps.
36. Centrifuge at 300 × *g* for 5 min and decant supernatant.
37. Resuspend cell pellet, add 2 mL of cold PBS and invert capped tubes to mix.
38. Centrifuge at 300 × *g* for 5 min and decant supernatant.
  a. Prepare a 1:1000 dilution of viability dye using eBioscience Fixable Viability Dye eFluor 780 in PBS.
39. Resuspend cell pellet and add 100 μL of viability dye mixture to each tube.
40. Incubate at 4°C for 30 min in the dark.
  a. Prepare antibody mix during the incubation by adding 1 μL of VioBright FITC Mouse Anti-Human CD271 (LNGFR) antibody into 49 μL of FACS buffer (1:50 dilution) per test.
41. Add 2 mL of FACS buffer and invert capped tubes to mix.
42. Centrifuge at 300 × *g* for 5 min and decant supernatant.
43. Resuspend cell pellet and add 50 μL of antibody mix to each tube.
44. Incubate at 4°C for 30 min in the dark.
45. Add 2 mL of FACS buffer and invert capped tubes to mix.
46. Centrifuge at 300 × *g* for 5 min and decant supernatant.

**CRITICAL:** Cells transduced with lentivirus must be fixed prior to sample acquisition by flow cytometry.

47. Resuspend cell pellet at add 100 μL of 2% PFA mix to each tube.
48. Incubate at 4°C for 1 h in the dark.
49. Add 2 mL of FACS buffer and invert capped tubes to mix.
50. Centrifuge at 300 × *g* for 5 min and decant supernatant.
51. Resuspend cell pellet and acquire samples by flow cytometry.

**Pause point:** Samples may be stored at 4°C for up to 5 days after fixation before sample acquisition by flow cytometry. However, we recommend acquiring samples within 24–48 h.

52. Decontaminate anything that has come into contact with the virus using 70% ethanol.

***Note:*** Minimize the production of aerosols when working in BSL2 conditions.

### Transduction of expanded NK cells with HER2 CAR lentivirus construct

**Timing: 3 days**

This step describes the process of transducing expanded NK cells with 3^rd^ generation LVs. Prior to NK cell transduction, determine the number of NK cells you need to transduce to perform downstream functional assays. In addition, you need to determine the amount of lentivirus needed to transduce desired number of wells at the desired MOI. Typically an MOI of 5 results in sufficient transduction efficiency. This is based on the LV titers typically obtained with our construct after titration on HEK293T cells. However, the MOIs used should be optimized depending on the size of your CAR lentiviral construct.

***Note:*** Primary NK cells should be expanded by co-cultured with irradiated K562 mb-IL21 feeder cells at a 2:1 ratio for at least 2 weeks before transduction.

53. A day before transduction, replenish expanded NK cells with irradiated K562 mb-IL21 cells at a 1:1 ratio.
  a. Seven days after the last replenishment, count NK cells with Trypan Blue.
  b. Resuspend the co-culture to a final concentration of 0.5 × 10^6^ cells/mL in complete RPMI media supplemented with 100 IU/mL of IL-2.
54. Plate 200 μL of the co-culture into a 96-well U bottom plate for a total of 1 × 10^5^ cells per well.
55. Incubate plate for 12–18 h at 37°C and 5% CO_2_.
56. 18–24 h after plating, centrifuge 96-well U bottom plate at 300 × *g* for 10 min.
  a. During the centrifugation, prepare the NK cell transduction media.
57. Carefully remove the supernatant from each well, and resuspend cells with 90 μL of NK cell transduction media using a multichannel pipette.

**CRITICAL:** Prepare fresh NK cell transduction media each time.

58. Place plate in the incubator (37°C and 5% CO_2_) while you prepare the viral suspension.

**CRITICAL:** Pre-warm the centrifuge to 32°C prior to thawing your virus so that you can perform the spinfection process as soon as possible.

59. Prepare viral suspension by diluting the stock with the appropriate amount of cold PBS to add 10 μL of viral suspension per well at an MOI of 5.

***Note:*** Thaw your lentivirus containing the CAR construct and the control vector on ice. If the lentivirus titre is <5 × 10^7^ TU/mL do not dilute in PBS.

Example: To calculate the amount of lentivirus needed to transduced 5 wells at an MOI of 5 use the following calculation (use virus titer obtained from the HEK293T titration):

((1 × 10^5^ × MOI × # of wells)/virus titer(TU/mL)) × 1000 = Total amount of virus needed (μL)

60. Once the virus suspension is prepared, obtain 96-well plate from the incubator and add 10 μL of virus suspension per well.
61. Add 10 μL of PBS to the non-transduced control wells.
62. Immediately centrifuge the plate at 1000 × *g* for 45 min at 32°C as the spinoculation step.

***Note:*** In our experience, we find that while spinoculation is not necessary for lentiviral transduction of human T cells, it does enhance the transduction efficiency of expanded NK cells. However, this step may be skipped depending on the size and titer of your lentiviral construct but should be optimized.

63. Gently resuspend the wells using a micropipette and incubate plate for 12–18 h at 37°C and 5% CO_2._
64. The next day, centrifuge the plate at 300 × *g* for 10 min.
65. Carefully remove the supernatant and resuspend cells to a final volume of 100 μL using complete RMPI media supplemented with 100 IU/mL of IL-2.
66. Three to four days after transduction, HER2 CAR-NK cells can be stained to assess HER2 CAR transgene expression.

### Assessing HER2 CAR expression by flow cytometry

**Timing: 3–4 h set up and staining; variable flow time**

This procedure describes how to assess expression of the HER2 CAR transgene after NK cell lentiviral transduction. The HER2 CAR transgene used in this study is under the EF1a promoter and the transduction marker is under the CMV promoter, the construct is described in Hammill et al. (2015).

67. Pool wells containing transduced HER2 CAR- and control NK cells (non-transduced and control vector transduced) into a 5 mL polystyrene tube.
68. Wash cells with 2 mL of complete RPMI media and centrifuge at 300 × *g* for 5 min.
69. Carefully discard the supernatant and count cells with Trypan Blue.
70. Transfer 100,000 cells of the HER2 CAR-, control vector- and non-transduced NK cells to a 96-well U bottom plate for staining with the surface stain cocktail.

**Table:** Surface stain master mix

Reagent	Dilution	Amount per test (μL)
Anti-CD56 BV421	1:20	2.5
Anti-CD3 APC-H7	1:20	2.5
Anti-IgG Fc PE	1:20	2.5
Anti-NGFR APC	1:20	2.5
FACS Buffer	N/A	40
**Total**		**50**

71. Prepare additional control wells using 100,000 cells per well.
  a. Plate one well with the HER2 CAR-NK cells HER2 Fc chimera protein control.
  b. Plate four wells with the non-transduced NK cells for the unstained, viability dye compensation and fluorescence minus one (FMO) control wells.

**Table:** Control well set up

	HER2 Fc chimera	CD56 BV421	CD3 APC-H7	IgG Fc PE	NGFR APC
Unstained	–	–	–	–	–
HER2 Fc chimera protein control	–	X	X	X	X
CD56 BV421 FMO	X	–	X	X	X
CD3 APC-H7 FMO	X	X	–	X	X

***Note:*** FMOs for the IgG Fc PE and NGFR APC can be included but the transgene positive gates should be set based on the fully stained non transduced NK cell control sample.

72. Resuspend the remainder of the cells to a final concentration of 1 × 10^6^ cells/mL in complete RMPI media supplemented with 100 IU/mL IL-2.
  a. Incubate at 37°C and 5% CO_2_ until functional assays.

***Note:*** Functional assays should be performed within 1–2 weeks after initial transduction as we have found transgene expression is downregulated over time and CAR-NK cells cannot be further expanded.

73. Spin the plate at 300 × *g* for 5 min to pellet the cells.
74. Remove supernatant and wash cells with 200 μL of PBS.
75. Spin the plate 300 × *g* for 5 min and discard PBS.
  a. Prepare a 1:1000 dilution of viability dye (Fixable Viability Stain 510) in PBS.
76. Add 100 μL of the viability dye and incubate at 4°C for 30 min in the dark.
  a. Prepare the HER2 Fc chimera protein mix during the incubation by adding 2.5 μL of recombinant ErbB2/HER2 Fc chimeric protein to 25 μL of FACS buffer per test.

***Note:*** You can also perform viability staining with Fixable Viability Stain 510 at 37°C for 7 min.

77. Spin the plate at 300 × *g* for 5 min and discard supernatant.
78. Wash cells with 200 μL of FACS buffer, spin the plate at 300 × *g* for 5 min and discard supernatant.
79. Add 25 μL of the HER2 Fc chimera protein dilution and incubate in the dark for 30 min at 20°C–22°C.
  a. Do not add HER2 Fc chimera protein to the extra HER2 CAR-NK cell control well.
80. Spin the plate at 300 × *g* for 5 min and discard supernatant.
81. Wash cells with 200 μL of FACS buffer, spin the plate at 300 × *g* for 5 min and discard supernatant.
82. Add 50 μL of the surface stain master mix and incubate 4°C for 30 min in the dark.

**Table:** Surface stain master mix

Reagent	Dilution	Amount per test (μL)
Anti-CD56 BV421	1:20	2.5
Anti-CD3 APC-H7	1:20	2.5
Anti-IgG Fc PE	1:20	2.5
Anti-NGFR APC	1:20	2.5
FACS Buffer	N/A	40
**Total**		**50**

***Alternatives:*** Anti-CD3 APC-H7 can be swapped out of this panel with Anti-CD3 PerCp-Cy5.5 and used at the same dilution, in that case you can use the fixable viability dye eBioscience Fixable Viability Dye eFluor 780 instead of Fixable Viability Stain 510.

83. Spin the plate at 300 × *g* for 5 min and discard supernatant.
84. Wash cells with 200 μL of FACS buffer, spin the plate at 300 × *g* for 5 min and discard supernatant.

**CRITICAL:** NK cells transduced with lentivirus must be fixed prior to sample acquisition by flow cytometry.

85. Add 100 μL of 2% PFA mixture and incubate at 4°C for 30 min in the dark.
86. Spin the plate at 300 × *g* for 5 min and discard supernatant.
87. Wash cells with 200 μL of FACS buffer, spin the plate at 300 × *g* for 5 min and discard supernatant.
88. Resuspend cells to 200 μL of FACS buffer.

**Pause point:** Samples may be stored at 4°C for up to 5 days after fixation before sample acquisition by flow cytometry. However, we recommend acquiring samples within 24–48 h.

***Note:*** Prepare single color compensation controls using compensation beads prior to sample acquisition by flow cytometry.

89. Transfer cells to appropriate tubes for flow cytometry and perform sample acquisition.

***Note:*** Ensure that HER2 CAR-NK cells have a minimum of 15% CAR transgene expression before functional assays (Troubleshooting 5).

### Flow cytometry-based HER2 CAR-NK cell cytotoxicity assay

**Timing: 1–3 h setup time; 5 h incubation time; 2 h staining time**

This step describes how to prepare target cells for the flow cytometry-based cytotoxicity assay and how to plate the effector and target cells. We recommend using 100,000–200,000 target cells but this number can be reduced to 50,000 cells. We recommend to include at least three Effector:Target (E:T) ratios in the cytotoxicity assay to obtain a killing curve.

**Figure 4:**
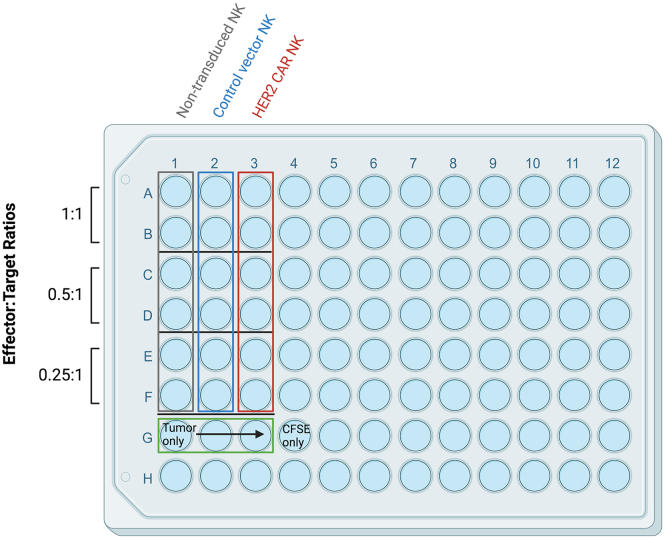
Example of a cytotoxicity assay plate layout comparing the *in vitro* anti-tumor function of non-transduced, control vector-, and HER2 CAR-transduced NK cells.

***Note:*** Due to the high susceptibility of SKBR3 breast cancer cells to expanded NK cell killing we chose the 1:1, 0.5:1 and 0.25:1 E:T ratios to assess *in vitro* anti-tumor function. The following protocol uses 100,000 target cells per well.

90. Label a 96-well plate layout with all of your conditions, follow Figure 4 as an example.

91. Prepare effector NK cells for plating.
  a. Transfer cells to a 15 mL falcon tube and centrifuge at 300 × *g* for 5 min.
  b. Carefully remove supernatant and count cells with Trypan Blue.
  c. Resuspend NK cells with complete RPMI media to a final concentration needed to add 100 μL of effector cells at the highest E:T ratio.

Example: If the highest E:T ratio is a 1:1, resuspend the effector NK cells to a final concentration of 1 × 10^6^ cells/mL in order to add 100,000 NK cells in 100 μL.

92. Set effector NK cells aside while you prepare the target cells.

***Optional:*** One day before the cytotoxicity assay, split SKBR3 target cells 1:2 to ensure high tumor cell viability on the day of the assay.

93. Harvest SKBR3 target cells by washing with 10 mL warm PBS and adding 5 mL of 1× Trypsin-EDTA (0.05%).
94. Neutralize trypsin by adding 3 times the volume of complete DMEM media.
95. Transfer cells to a 50 mL falcon tube and centrifuge at 300 × *g* for 5 min.
96. Discard supernatant and wash cells with 20 mL of PBS.
97. Centrifuge at 300 × *g* for 5 min.
98. Discard supernatant and count cells with Trypan Blue.
  a. Resuspend cells with a low volume for counting since they will need to be highly concentrated for CFSE-labeling.

**CRITICAL:** The viability of the target tumor cells should be >80% on the day of the assay.

***Alternatives:*** Other fluorescent cell labelling reagents can be used such as Cell Trace Violet (Thermo Fisher).

99. Resuspend total amount of SKBR3 cells needed for the cytotoxicity assay at a concentration of 10 × 10^6^ cells/mL in PBS in a 50 mL falcon tube.
100. Prepare 5 μM CFSE solution.
  a. Prepare a series of three 15 mL falcon tubes labeled A, B, C. Add 990 μL DMSO to Tube A; Add 2.70 mL PBS to Tube B; Add 9 mL of PBS to Tube C.
  b. Add 10 μL of CFSE (50 mM) stock into Tube A and vortex.
  c. Take 300 μL from Tube A and add it to Tube B. Vortex.
  d. Take 1 mL from Tube B and add it to Tube C. Vortex.

***Note:*** Working concentration of CFSE can be prepared earlier but should be stored at 4°C protected from light before use.

101. Add an equal volume of CFSE mixture from Tube C to the resuspended target cells.

Example: If a total of 10 million target cells are resuspended in 1 mL of PBS, add 1 mL of CFSE mixture from Tube C.

102. Immediately vortex at a low-med speed, making sure cells are properly mixed.
103. Incubate the CFSE-labeled cells at 37°C for 15 min.

***Note:*** Protect the CFSE-labeled cells from light using tinfoil.

104. After incubation, quench the unbound CFSE by topping up the 50 mL falcon tube with 10% FBS-PBS for 10 min at 20°C–22°C in the dark.
105. Centrifuge at 300 × *g* for 5 min.

***Note:*** The CFSE-labeled cell pellet can look slightly green at this point.

106. Wash cells with 20 mL of PBS and centrifuge at 300 × *g* for 5 min.
107. Discard supernatant and resuspend cells in a low amount of **complete RMPI media.**
108. Count cells using Trypan Blue and resuspend to 1 × 10^6^ cells/mL with complete RPMI media.
  a. The target cells are now ready to be plated.
109. Plate the effector NK cells according to each E:T ratio and top up wells to a total volume of 100 μL with complete RPMI media.
  a. 1:1 E:T; Add 100 μL of NK cells.
  b. 0.5:1 E:T; Add 50 μL of NK cells and 50 μL of complete RPMI media.
  c. 0.25:1 E:T; Add 25 μL of NK cells and 75 μL of complete RPMI media.
110. Prepare the tumor only control wells by adding 100 μL of complete RPMI media.
111. Use a multichannel pipette to add 100 μL of CFSE-labeled target cells to wells containing the effector NK cells and the tumor only wells.
  a. Plate one extra well with the target cells to use as the CFSE-only control well to adjust voltages during sample acquisition.
112. Incubate cytotoxicity assay 96-well plate at 37°C and 5% CO_2_ for 5 h.
113. Spin plate at 300 × *g* for 5 min and discard supernatant.

***Optional:*** Carefully collect ∼150 μL of the cell-free supernatants using a multichannel pipette and transfer to a new 96-well U or V bottom plate. Place plate in −20°C freezer for cytokine analysis.

114. Wash wells using 200 μL of PBS, spin plate at 300 × *g* for 5 min and discard supernatant.
  a. Prepare a 1:1000 dilution of viability dye using eBioscience Fixable Viability Dye eFluor 780 in PBS.

**CRITICAL:** CFSE has an excitation of 492 nm and emission of 517 nm, stain the cytotoxicity assay using a fixable viability dye which does not overlap. We use eBioscience Fixable Viability Dye eFluor 780 which has an excitation of 633 nm and emission of 780 nm. In our experience, this panel does not require compensation or FMO controls.

115. Add 100 μL of fixable viability dye and incubate at 4°C for 30 min in the dark.
116. Spin plate at 300 × *g* for 5 min and discard supernatant.
117. Wash wells with 200 μL of FACS buffer, spin plate at 300 × *g* for 5 min and discard supernatant.
118. Add 100 μL of 1% PFA mixture and incubate at 4°C for 1 h in the dark.
119. Spin plate at 300 × *g* for 5 min and discard supernatant.
120. Wash wells with 200 μL of FACS buffer, spin plate at 300 × *g* for 5 min and discard supernatant.
121. Resuspend plate with 200 μL of FACS buffer.

**Pause point:** Samples may be stored at 4°C for up to 5 days after fixation before sample acquisition by flow cytometry. However, we recommend to acquire samples within 24–48h.

122. Transfer samples to appropriate tubes for flow cytometry and perform sample acquisition.

## Expected outcomes

**Figure 5:**
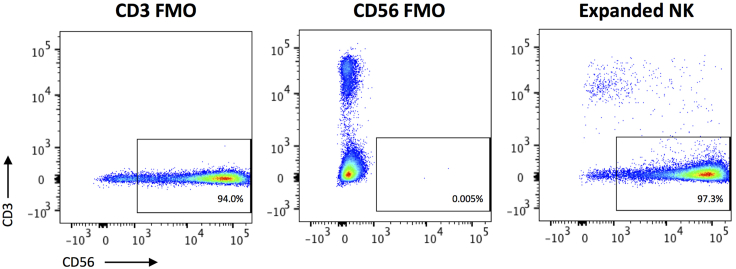
Purity of CD3-CD56^superbright^ expanded NK cells after 3 week expansion The CD3-negative and CD56-positive gates were set using the corresponding FMOs. Samples were acquired using the LSRFortessa Flow Cytometer and analyzed with FlowJo Software.

The expected purity of the expanded CD3-CD56^superbright^ NK cells is demonstrated in Figure 5.

**Figure 6:**
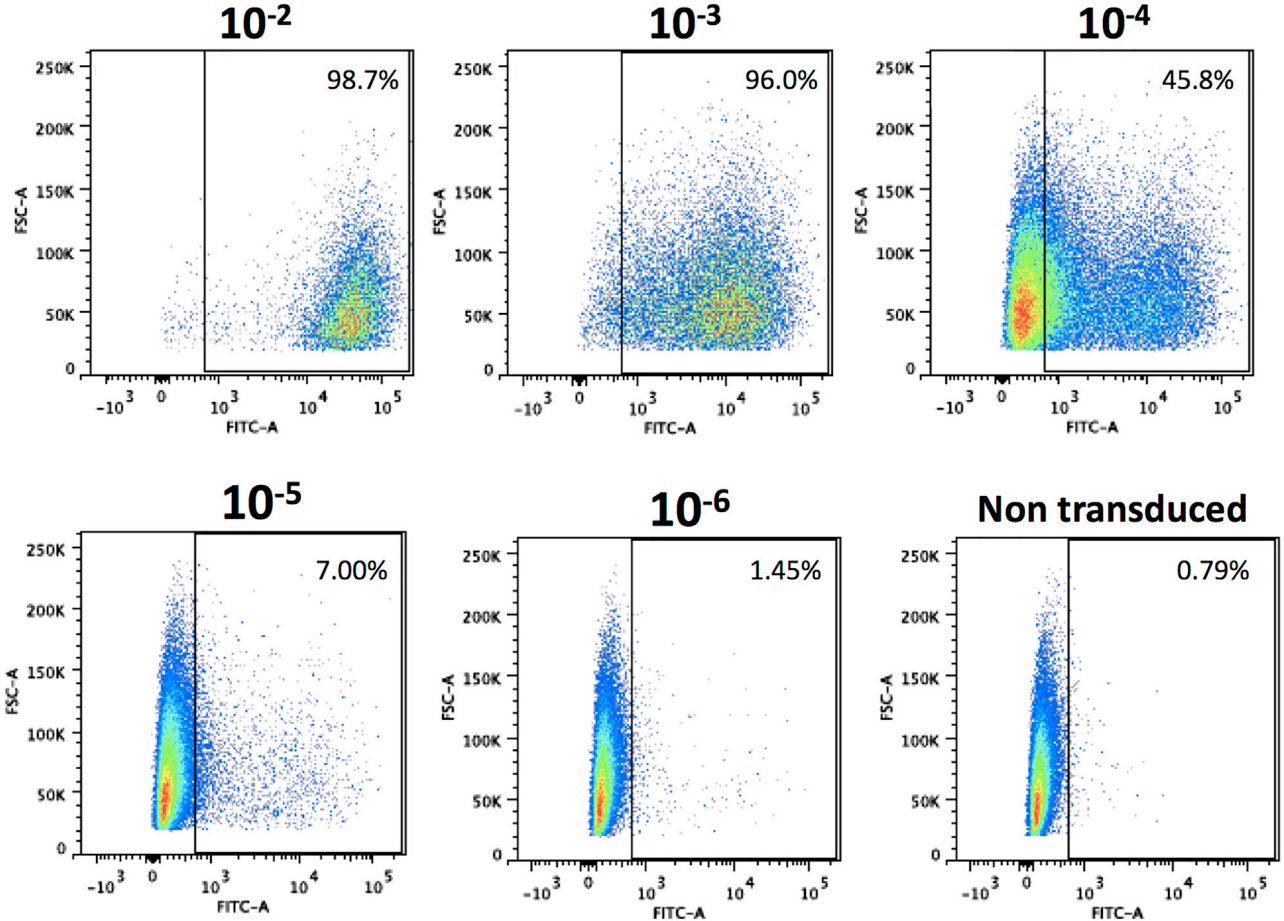
Example flow plots of transgene expression on HEK293T cells 72h after lentiviral titration To determine the lentivirus titer use the formula: Titer (TU/mL)= ((30,000 × % Transgene-positive cells X Dilution factor)/100). Set the transgene-positive gate on the sample that was stained but did not obtain any lentivirus and use the dilution factor which results in >10% transgene-positive cells. Samples were acquired using the LSRFortessa Flow Cytometer and analyzed with FlowJo Software.

The expected titer for the lentivirus containing the HER2 CAR construct in this study is between 3 × 10^7^ and 2 × 10^8^ TU/mL, use Figure 6 as a guide to set the transgene-positive gates after the lentiviral titration.

**Figure 7:**
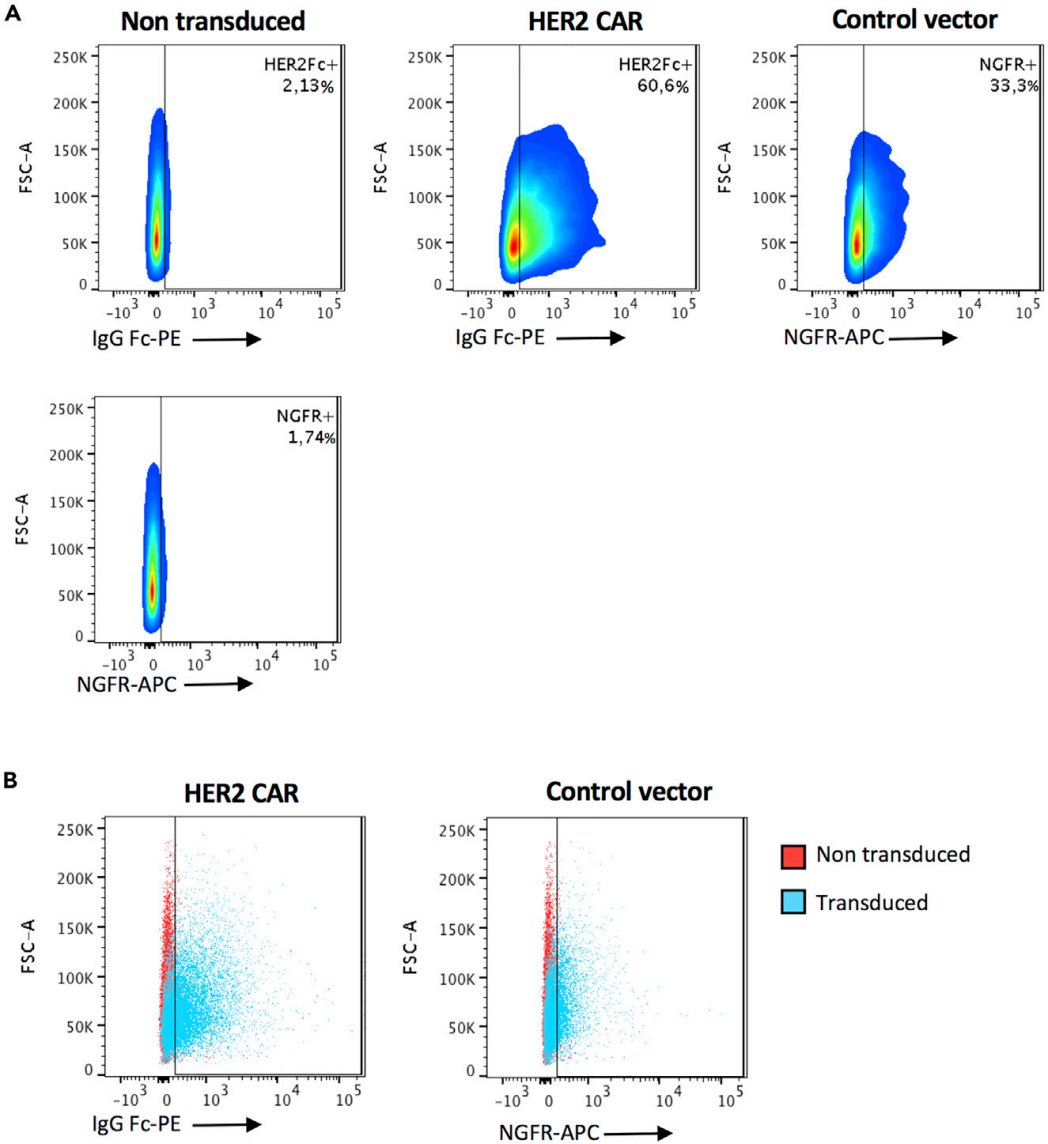
Example flow plots of transgene expression on HER2 CAR- and control vector-transduced expanded NK cells (A) Percent transgene expression in the non transduced, HER2 CAR-, and control vector transduced NK cells. (B) Extent of transgene expression in the transduced NK cells compared to the non transduced control. Samples were acquired using the LSRFortessa Flow Cytometer and analyzed with FlowJo Software.

The transduction efficiency of the CAR construct can vary between donors, generally we obtain at least 15% transduction efficiency, see Figure 7 for an example.

**Figure 8:**
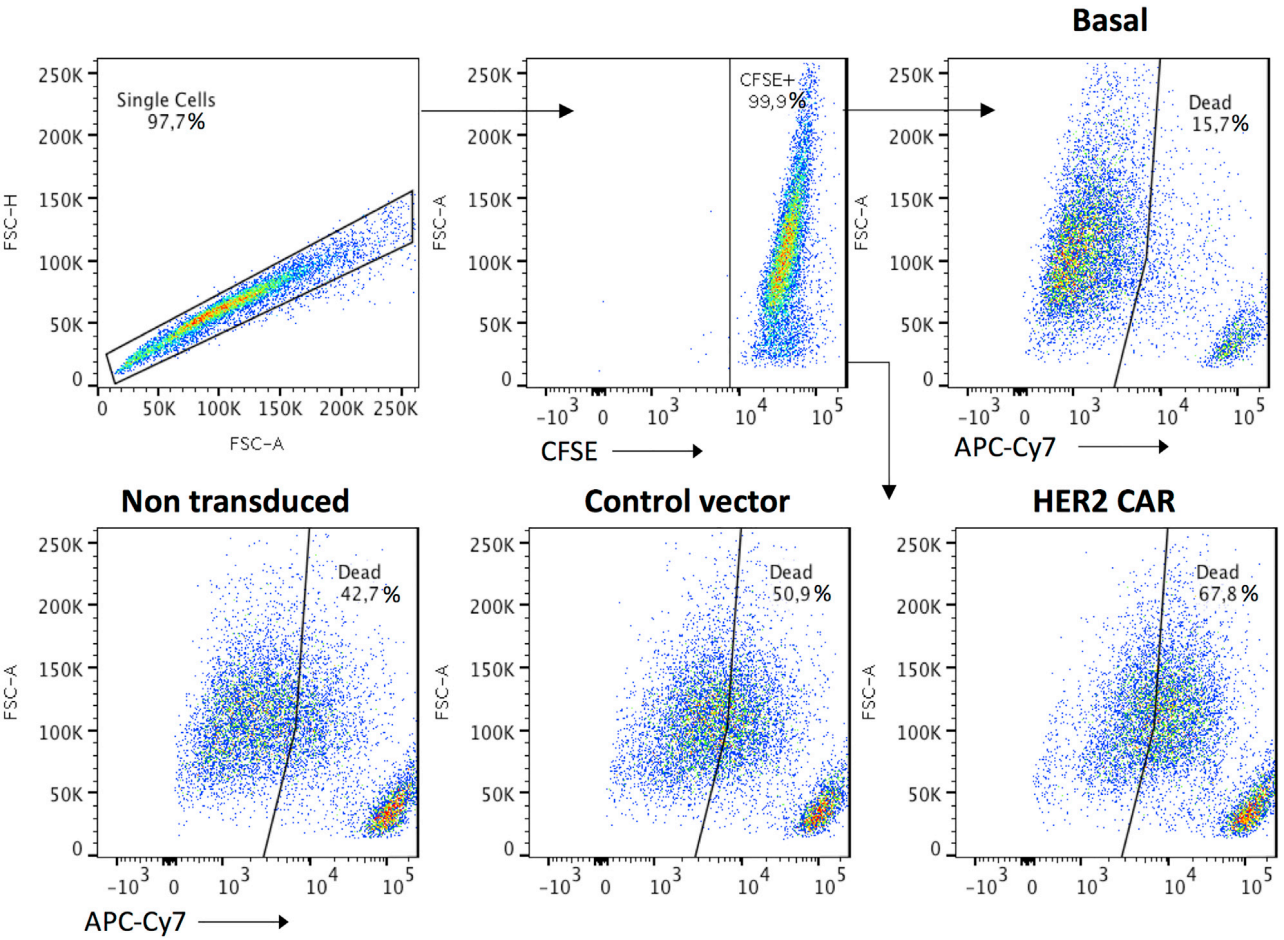
Example flow plots of *in vitro* cytotoxicity assay Singlets are discriminated (FSC-H v. FSC-A) and CFSE-positive tumor cells are gated. Create a dead cell gate (APC-Cy7 positive) based on the samples that only contain tumor cells and no effector cells (basal). Apply dead cell gate to the non-transduced, control vector, and HER2 CAR-transduced expanded NK cell samples. Samples were acquired using the LSRFortessa Flow Cytometer and analyzed with FlowJo Software.

To determine the percent-specific lysis of the control and HER2 CAR-expressing effector NK cells first determine the percentage of dead target cells using the gating strategy defined in Figure 8. Calculate percent-specific lysis using the following formula:

% specific lysis = ((% tumor cell death – % basal tumor cell death)/(100 – % basal tumor cell death)) × 100

## Limitations

The following protocol can be used to generate human CAR-NK cells with transduction efficiencies ranging between 15 and 80%. However, a main limitation is that transgene expression is downregulated over time thus functional assays should be performed within one to 2 weeks of CAR-NK cell generation. Protocols that use retrovirus instead of lentivirus or include sequences that prevent promoter silencing in their CAR plasmid may yield CAR-NK cells with stable transgene expression (Colamartino et al., 2019; Liu et al., 2020). Additionally, other promoters such as SFFV may lead to greater transduction efficiencies however we only used the EF1alpha and CMV promoters in our studies.

Another limitation is that the transduction protocol has been optimized for use with human NK cells that have been expanded using genetically engineered K562 feeder cells. This protocol may require further optimization for the transduction of cytokine activated NK cells which are typically more difficult to transduce. However, we have also used the protocol to successfully generate CAR-NK cells from the NK-92 human NK cell line as described in Portillo et al. (2021).

## Troubleshooting

### Problem 1

Low viability and slow expansion of NK cells from starting PBMCs (Before you begin steps 14–19).

### Potential solution

Using fresh PBMCs as opposed to cryopreserved PBMCs to expand the NK cells usually yields a higher viability and expansion rate. In addition, changing the media and adding IL-2 every 2 days is crucial to maintain a good viability of the rapidly proliferating NK cells in culture. Do not use IL-2 aliquots that have been thawed and stored at 4°C for longer than 2–3 days.

### Problem 2

A non-specific CD3+ T cell population has expanded in the expanded NK cell culture (Before you begin step 19).

### Potential solution

Perform a CD3+ positive selection using commercial isolation kits to remove the contaminating CD3+ T cells and continue replenishing the culture. Additionally, CD3+ cells can also first be depleted from the starting PBMCs using an immunomagnetic CD3+ isolation kit. If the CD3+ T cell population continues to expand, use PBMCs from a different donor.

### Problem 3

Low lentiviral yield after ultracentrifugation (step 25).

### Potential solution

The transfer vector containing your desired transgene might be too large to efficiently generate 3^rd^ generation lentivirus using this protocol. We find that lentiviral titer and LV production efficiency decreases as the size of the transgene packaged in the LV increases. In this case, the 2^nd^ generation lentivirus packaging system could be used since all the packaging genes are encoded within only one vector as opposed to two like in the 3^rd^ generation system. Using the 2^nd^ generation system has shown to increased viral yields 50-fold (Cribbs et al., 2013). However, 3^rd^ generation LVs are preferred for clinical applications due to the added biosafety aspect.

### Problem 4

Low lentiviral titer (<1 × 10^7^ TU/mL) is obtained after lentiviral production (Titer will be determined after step 51).

### Potential solution

If the LV titer is lower than 1 × 10^7^ TU/mL after ultracentrifugation there was an issue during LV production. Ensure that HEK293T cells have a viability between 90 and 95% at every plating step. We recommend that HEK293T cells are no more than 80% confluent during the transfection step to prevent cells lifting off the plate during the media change the next day as this can highly impact viral titer. It is also critical that your starting plasmids have high purity for successful HEK293T transfection. Using commercially available plasmid isolation kits such as the PureLink™ HiPure Plasmid Maxiprep Kit (Thermo Fisher) is recommended to obtain high purity. You may also need to optimize the amount of plasmids used for the transfection.

### Problem 5

A low transduction efficiency (<15%) is obtained after NK cell transduction (Transduction efficiency will be determined after step 89).

### Potential solution

In our experience we find that lentivirus preps with a titer >3 × 10^7^ TU/mL are necessary to yield CAR-NK cells with high transgene expression. In addition, we have found that using expanded NK cell cultures that have not been expanded beyond 8 weeks typically yield higher transduction efficiencies. The MOIs needed to transduce NK cells may also need to be optimized. The CAR lentiviral construct used in this study is ∼10 Kb, and we find MOIs 3–8 are sufficient to yield >15% transduction efficiencies. However, higher MOIs may be needed for larger lentiviral constructs. We also find that transgene efficiency can also be donor dependent.

## Resource availability

### Lead contact

Further information and requests for resources and reagents should be directed to and will be fulfilled by the lead contact, Dr. Ali A. Ashkar (ashkara@mcmaster.ca).

### Materials availability

The study did not generate new unique reagents.

## Data Availability

No data or code was generated or analyzed in this protocol.
